# The Mediating Role of Academic Passion in Determining the Relationship Between Academic Self-Regulation and Goal Orientation With Academic Burnout Among English Foreign Language (EFL) Learners

**DOI:** 10.3389/fpsyg.2022.933334

**Published:** 2023-02-02

**Authors:** Siros Izadpanah

**Affiliations:** Department of English Language Teaching, Zanjan Branch, Islamic Azad University, Zanjan, Iran

**Keywords:** academic motivation, academic passion, academic self-regulation, academic burnout, goal direction, learning

## Abstract

One of the most significant current discussions in educational psychology is academic passion. This research aimed to investigate the mediating role of academic passion in the relationship between goal orientation and academic self-regulation with students’ academic burnout. However, so far, there has been little discussion about these variables together. The statistical population of the present study includes all undergraduate English students of Islamic Azad University, Tabriz, whose strength is equal to 598 people, and the sample size is determined by the convenience sampling method and Cochran’s formula (248 participants). Data collection methods in this study include four questionnaires of the academic passion; goal orientation; self-regulatory and academic burnout. The Pearson test results showed that the correlation coefficient of academic burnout with goal orientation, self-regulatory, and academic passion is statistically significant at 0.05%. These variables are inconsistent with academic burnout, and with increase in these variables, academic burnout significantly decreases. The correlation coefficient of goal orientation with self-regulatory variables and academic passion is statistically significant at the level of 0.05%. These variables are consistent with goal orientation, self-regulated learning, and academic passion, and with increase in the aforementioned variables, they significantly rise. Goal-oriented and self-regulated goal orientation can predict academic passion. Academic passion has a direct and significant relationship with academic self-regulation and goal orientation. Finally, the obtained fit indices of the general model have the desired fit with the collected data. The implication is that academic passion plays an important role in creating a positive learning environment that will lead to effective learning and teaching. It may influence student learning. Passion inspires and stimulates, and teaching with passion promotes learning.

## Introduction

Students are the cornerstone of the education system in every society, and one of the most important tasks of this system is to invest in learners’ achievements. Researchers are constantly attempting to identify variables influencing learners’ academic performance (Sharififard et al., 2014; Fernandez-Perez and Martin-Rojas, 2022). There has been much stress on students due to the coronavirus and extracurricular classes in recent years. On the other hand, universities and educational institutions do not meet the psychological needs of students. Therefore, students gradually suffer academic burnout, a variable that researchers in this field have considered as one of the factors affecting academic achievement (Lin and Huang, 2014).

Freudenberger (1974) first defined burnout as a pervasive general phenomenon resulting from a person’s interaction with the environment that leads to a loss of motivation, passion, energy, and reduced performance. Burnout is commonly regarded as a three-dimensional disorder, including emotional exhaustion, personality decline, and decreased self-efficacy. Personality decline refers to pessimistic or overly reluctant negative responses to other people in the workplace that indicate an interpersonal component of burnout. Burnout in education has various symptoms, including lack of enthusiasm for the subject, inability to continue attending the class, lack of participation in-class activities, inadequacy and inefficiency in university activities, and failure to learn the subject. Also, the sense of tiredness due to educational needs, sensitivity, worthlessness, passion, apathy toward university, and inadequacy can be considered the most important symptoms (Salmela-Aro and Tynkkynen, 2012; Goldan et al., 2022).

Another factor that can be related to learning is academic passion. Students’ academic passion is a variable that is vital to learning to the extent that it is mentioned as an essential learning factor. Academic passion refers to the amount of energy a learner spends doing their academic work, as well as the amount of effectiveness and efficiency achieved. Academic passion has been mentioned as a psychological capital of students and their direct efforts to learn, acquire skills, and desire to improve their level of success, which can lead to effective participation in university activities, participation in-class activities, adaptation to university culture, and appropriate relationship with teachers and other students (Chichekian and Vallerand, 2022).

One of the most prominent issues in self-regulation is the issue of academic goals and goal orientations. In general, goal orientation theories have been developed to explain the behavior of progress, especially in educational settings and in learning and learners’ performance in completing academic assignments. Goal orientation is one of the psychological variables that Kumar et al. (2022) consider a coherent model of one’s beliefs that leads to adopting different ways of dealing with, engaging in, and responding to learning situations.

Academic self-regulation is another variable that can play a role in the relationship between academic passion and academic burnout that has a mediating role. Bandura’s social cognitive theory provided an appropriate theoretical framework for developing self-regulated learning. According to contextual factors and behaviors, each variable provides the necessary opportunity to control student learning. Self-regulation means that students can design, control, and direct their learning process. They tend to learn and evaluate the whole learning process and think that this process, in turn, reduces student fatigue and burnout (Nikos and George, 2005). Self-regulation is the process by which persons monitor their behavior; judge their behavior according to their standards, goals, and criteria; and as a result, adjust their behavior (Sahaghi and Moridi, 2015).

In education, students develop self-confidence in different ways, and those who are more confident are more involved in academic activities. These beliefs also affect persons’ way of thinking, feeling, motivating, or behaving. A strong sense of academic self-regulation and goal orientation improves positive individual attitudes and better student participation in academic activities (Wang et al., 2021; Alt et al., 2022; Janke, 2022).

The present study intends to examine the structural model of the relationship between goal orientation and academic self-regulation with academic burnout while exploring the direct effects to answer the question of whether academic passion plays a mediating role in the relationship between goal orientation and academic self-regulation with academic burnout?

## Review of Literature

### Academic Passion

Academic passion can be associated with academic self-regulation. Students’ academic passion is a variable that is vital to learning to the extent that it is mentioned as an important learning factor. Academic passion refers to the amount of energy a learner spends doing their academic work, as well as the amount of effectiveness and efficiency achieved (Lavasani et al., 2011; Mousavi, 2015; Zaregar et al., 2017; Roberts et al., 2022).

Yang et al. (2022) have mentioned academic passion as students’ psychological capital and their direct efforts to learn and acquire skills, and the desire to improve their level of success, which can lead to effective participation in school activities, participation in-class activities, adaptation to school culture, and a good relationship with teachers and other students.

In various humanities books, “motivation” is used, instead of “passion.” It has been said that the keyword of motivation in multiple sources is that human actions are of two types: One is voluntary, and the other is motivational. Voluntary actions are actions that a person performs consciously and with order and intention. Dini et al. (2022) doubt the existence of powers, such as memory, accuracy, an association of meanings, and thinking. First, they considered these divisions unlimited, and second, they assumed that motivational activities are a kind of relationship between neurons in the nervous system. They say that motivational actions do not excel in practice and repetition, and also, the more specific they are, the less generalizable they become. Education can be considered an organization of humanization, growth, and development of society (Seif and Ali Akbar, 2003; Shokri et al., 2008; Zhao et al., 2021). One of the most important factors that play a crucial role in education is the student. In other words, society, especially educational systems, is interested and concerned about the fate of the individuals, their successful growth and development, and their position in society and keeps expectations on the individuals in various aspects, including cognitive dimensions and the acquisition of skills and abilities, as well as in personality and emotional and behavioral dimensions as it should be developed and exalted (Zimmerman, 2008; Barimani et al., 2021).

Students’ passion for education is another important variable that is vital for learning, and it is considered an important harbinger of learning. This variable is also the focal point of most theories related to academic failure (Zhao et al., 2021). Academic passion refers to the amount of energy a learner spends doing their work, as well as the amount of effectiveness and efficiency achieved (Bélanger and Ratelle, 2021). Students who are eager to study pay more attention to the issues and topics they are learning, show more commitment to university rules and regulations, avoid inappropriate and undesirable behaviors, and perform better on examinations. Academic passion includes three dimensions: self-absorption, strength, and dedication. A prominent feature of attraction that refers to focusing and immersing oneself in academic activities is that time passes quickly for the learner so that the person does not recognize the passage of time and can hardly leave work. The strength dimension refers to high levels of power and flexibility of the pervasive mind when performing tasks in which students show significant effort in completing their assignments. The third component of academic motivation is devotion to academic activity, which is characterized by a severe personal–psychological conflict with academic duties. This dimension is a kind of academic attachment and a degree to which a person is psychologically dependent and committed to his academic affairs (Schell, 2022).

A study by Nishikawa et al. (2022) shows that the behavioral component alone played a significant role in the predictive equation among its components. Students who attend classes regularly focus on learning topics, adhere to school rules, score high overall, and perform better on standardized academic achievement tests.

Huéscar Hernández et al. (2020) state that desire for school is defined as an adaptation to school and participation in it. The two factors responsible for needs are participation in core and extracurricular activities. Tang et al. (2021) stated in a study that passion is characterized by academic and social components, which play a role in academic adjustment. Academic passion also includes students’ attitudes toward school and their ability to meet performance expectations.

Another variable is goal orientation. One of the primary and noteworthy factors in developing educational programs is determining and specifying the goals that educational activities are selected and arranged to achieve (Kumar et al., 2022). In addition to accomplishing a job or task as a common link, goals may also be an evaluation criterion. In addition to guiding the action, they also regulate energy expenditure and are the primary source of motivation (Ataee Far and Shaghaghi, 2011). Direction means what individuals ultimately want to achieve, that is, the long-term goal; it means looking far; but goal means the place where individuals do to reach the specified orientation, to get it in the present stage; the goal must always be visible and in the direction of human orientation. Not having the right goal leads to confusion and conflicting planning, and not having the right goal (if there is a direction) leads to unrealistic and impractical idealism. With this account, first, they have to specify the desired direction, set a clear and achievable goal for each stage, and move according to it. Suralaga et al. (2021) conducted a study entitled the role of moral self-regulation in mediating the effect of goal orientation on academic integrity. The results showed that the students confirmed the learning goals or mastery goals and social goals on functional tendency goals and functional avoidance goals. One of the most prominent issues in the field of academic self-regulation is the issue of goal orientation. In general, goal orientation theories have been developed to explain the behavior of progress, especially in educational settings and in the field of learning, as well as learners’ performance in completing academic assignments. Goal orientation is one of the psychological variables that Emiz (1992) considers as a coherent model of one’s beliefs that leads to different ways of dealing with, engaging in, and responding to learning situations. This orientation in educational situations indicates a person’s motivation to study and therefore affects their desires, actions, and responses in learning situations. Goal orientation does not only include the individual’s intentions and reasons for progress but also reflects the type of criteria by which individuals judge their performance (Cao et al., 2022).

### Academic Self-Regulation

Academic self-regulation is another variable that can play a mediating role in the relationship between academic passion and academic burnout. Bandura’s social cognitive theory provided an appropriate theoretical framework for developing self-regulated learning, according to which contextual factors and behaviors in each individual provide the necessary opportunity to control student learning. Self-regulation means that students can design, control, and direct their learning process (Nikos and George, 2005). They tend to learn and evaluate the whole learning process, and think that this process, in turn, reduces student fatigue and burnout. Self-regulation is how persons monitor their behavior; judge their behavior according to their standards, goals, and criteria; and adjusts their behavior. Research has shown that internally motivated learners engage in tasks more consistently and deeply and make higher progress than externally motivated to learn. Theoretically, having an inner motivation to learn and effective cognitive strategies and self-regulation is essential academic success factors. Students with high self-regulation attain more academic success and, therefore, more academic motivation (Duru and Balkıs, 2017; Anthonysamy and Choo, 2021; Öztürk and Çakıroğlu, 2021). In this sense, self-regulation is related to the rate of academic burnout (Kljajic et al., 2017).

Abbasi and Pirani (2015) in a study entitled he relationship between self-regulated learning and goal orientation with academic hope in first-grade female high-school students in Marvdasht city concluded that there is a significant direct relationship between self-regulated learning and goal orientation with academic hope. Self-regulation and goal orientation can predict hope for education.

Akhlaghi et al. (2015) conducted a study entitled to evaluate the effectiveness of multidimensional object-oriented interventions on task value, mastery of goal orientation and academic self-regulation. The results showed the effectiveness of a multidimensional cognitive–motivational intervention on the components of internal goal orientation and self-regulation of students in the experimental group, but the difference between the control and experimental groups in the component of task value was not significant. Multidimensional motivational–cognitive interventions increase the self-regulatory component and the formation of the internal goal orientation component in students.

### Burnout

One factor that affects students’ academic performance and has recently been studied in universities is academic burnout (Lee and Ashforth, 1996; Asikainen et al., 2022). Burnout is a state of mental and emotional fatigue that results from chronic stress syndromes, such as weight, stress and time constraints, and lack of resources needed to perform the assigned tasks (Naami, 2009; Sharififard et al., 2014; Jagodics and Szabó, 2022). The concept of burnout has also been extended to educational situations, which is referred to as academic burnout. Burnout is a feeling of tiredness due to academic demands and requirements, having a pessimistic sense and not being interested in homework, and feeling unworthy. It can reduce the level of energy required to perform cognitive tasks with learning, as well as the ability to focus on existing cognitive resources (Zhang et al., 2007; Pourkarimi and Mobinrahni, 2018).

Studies on the relationship between academic passion and academic burnout showed a negative correlation between academic passion and burnout (Lyndon et al., 2017; Fernandez-Perez and Martin-Rojas, 2022). Research has also shown that decreased academic passion causes depression and academic burnout. By examining the signs and symptoms of academic burnout, it can be recognized that the feeling of inability to learn what is known in the educational psychology literature as academic passion is one of the predictors of academic burnout (Sharififard et al., 2014; Zaregar et al., 2017; Jagodics and Szabó, 2022). Academic passion refers to individuals’ beliefs about their abilities to do academic work and is related to persons’ sense of power to learn and master the course content. Students with academic passion have more academic involvement and feel less academic burnout (Salmela-Aro and Tynkkynen, 2012). Consequently, it can be said that there is a relationship between goal orientation and academic burnout. Academic burnout consists of emotional fatigue, apathy, and academic inefficiency. In explaining this relationship, we can say people with less emotional exhaustion are more interested in affecting their self-regulation. Burnout causes a loss of energy and depletion of human resources, and impacts motivation and cognition. Self-regulation also affects cognition, motivation, and energy management. Thus, academic burnout and self-regulation can affect each other.

In addition to academic goal orientation, academic self-regulation is another variable that can mediate the relationship between academic passion and academic burnout. Bandura’s social cognitive theory provided an appropriate theoretical framework for developing self-regulated learning, according to which contextual factors and behaviors in each individual provide the necessary opportunity to control student learning.

The present study intends to answer the question of the relationship between goal orientation and academic self-regulation with academic burnout by examining the structural model of the relationship between goal orientation and academic self-regulation with academic burnout. Does academic passion play a mediating role?

### Research Hypotheses

Response: It was added.

Hypothesis No. 1: Goal orientation has a negative and significant effect on the academic burnout of English language undergraduate students in Tabriz.

Hypothesis No. 2: Self-regulation has a negative and significant effect on the academic burnout of English language undergraduate students in Tabriz.

Hypothesis No. 3: Goal orientation has a negative and significant effect on academic burnout with the mediating role of the academic passion of undergraduate English students in Tabriz.

Hypothesis No. 4: Self-regulation has a negative and significant effect on academic burnout with the mediating role of the academic passion of English language undergraduate students in Tabriz.

### Method

#### Design of the Study

The method of the present study is descriptive (non-experimental), and the correlational research design is structural equation modeling. This study examines the relationships between variables in a causal model. The statistical population of this study included students at Islamic Azad University, Tabriz, in the academic year 2021–2022 who were studying at the university.

#### Instruments

##### Burnout Questionnaire

The burnout questionnaire was developed by Berso et al. (1997). This questionnaire measures three areas of academic burnout, namely, academic fatigue, academic apathy, and academic inefficiency. The mentioned questionnaire has 51 items that have been graded by the subjects with the Likert grading method of one degree from disagreeing entirely to agree strongly. The reliability of the questionnaire was reported by using the internal consistency method (Cronbach’s alpha), and based on the subscales, 0.70 for academic fatigue, 0.82 for academic apathy, and 0.75 for academic inefficiency (Durak et al., 2022). In addition, the validity of the questionnaire was calculated by the confirmatory factor analysis method, which reported compliance matching indices 1, incremental fit indices (IFI) 2, and root mean squares of approximation error 3 (Han et al., 2022). In the present study, Cronbach’s alpha is based on subscales: 0.69 for academic fatigue, 0.72 for academic apathy, and 0.65 for academic inefficiency. In addition, the validity of the questionnaire was calculated by confirmatory factor analysis, which reported that the matching fit indices (CFI), IFI, and root mean square error approximation index (RMSEA) were favorable. In Abargooi (2010) study, Cronbach’s alpha calculated for the whole questionnaire was 0.85, and for the areas of emotional fatigue, pessimism, and academic inefficiency were 0.77, 0.82, and 0.66, respectively. This questionnaire has had a good validity in Marzooqi and Heidari (2013) study.

#### Academic Self-Regulation Questionnaire

The 14-item questionnaire of Bouffard-Bouchard et al. (1995) is a self-regulatory assessment instrument based on Bandura’s social cognitive theory. Questions are on the Likert scale and measure two factors of cognitive and metacognitive strategies (Kadivar, 2001). The scoring method using the Likert scale is strongly agree (score 5) to disagree strongly (score 1), and questions 5, 13, and 14 are reversed. Kadivar (2001) has studied the validity and reliability of this instrument. The construct validity of this questionnaire was reported to be optimal by using correlation coefficients, and factor analysis of correlation coefficients between the questions of the questionnaire and Cronbach’s alpha coefficient for measuring internal consistency was 0.08. Based on this, it can be said that this questionnaire can predict the actual scores of the participants. Zimmerman and Martinez-Pons (1990) have examined and reported this instrument’s standard and convergent validity to the desired extent. In the present study, Cronbach’s alpha test was used to evaluate the instrument’s reliability, which obtained values of 0.81.

#### Goal Orientation Questionnaire

The scale was developed by Elliot and McGregor (2001). This questionnaire has 12 items and consists of four components and measures the goal of progress with a five-point Likert scale (from very low to very high). Goal orientation is a way to judge one’s worthiness and express emotions, attributes, and beliefs about different situations. In this study, goal orientation is the score that respondents give to the 12-item questions of the goal orientation questionnaire. In Mushtaqi et al. (2012), the validity of the questionnaire has been confirmed by seven experts.

#### Questionnaire Reliability

Reliability or reliability of an instrument is its degree of stability in measuring everything measured, that is, the extent to which the measuring instrument gives the same results in the same conditions (Sarmad et al., 2011).

##### Academic Passion

The standard questionnaire of academic passion has been designed and developed by Fredricks et al. (2004). The questionnaire has 15 items and measures academic passion with a five-point Likert scale (never forever), with questions, such as “I pay attention in class; I just pretend to be active when I’m in class.” Questionnaire components: questions 1, 2, 3, and 4 related to the behavioral passion subscale; and questions 5, 6, 7, 8, 9, and 10 related to emotional desire; and questions 11, 12, 13, 14, and 15 also associated with the subscript “Is the scale of cognitive passion.” Bagheri et al. (2019) examined the validity and reliability of the questionnaire.

#### Sample Size Information

The statistical population of the present study includes all undergraduate English students of Islamic Azad University, Tabriz, whose strength is equal to 598 people, and the sample size is determined by the convenience sampling method and Cochran’s formula for a limited population as follows:


$$n=\frac{N\,Z_{\frac{a}{2}}^{2}\times p\,\left(1-p\right)}{N\,e^{2}+Z_{\frac{a}{2}}^{2}\times p\,\left(1-p\right)}=\frac{598+1.96^{2}\times 0.25}{598\times 0.05^{2}+1.96^{2}\times 0.25}=233.90\approx 234$$

To use structural equations, factor validity is required, the results of which are presented in Table 1:

**TABLE 1:** Kaiser-Meyer-Olkin Measure of sampling adequacy (KMO) and Bartlett’s test to evaluate the adequacy of sampling and data correlation.

Variables	KMO	Bartlett’s test of sphericity	df.	Sig.
Academic burnout	0.804	1151.057	105	0.000
Goal orientation	0.922	2193.859	66	0.000
Self-regulatory	0.955	2800.521	91	0.000
Academic passion	0.902	1605.013	45	0.000

According to Table 1, the Kaiser-Meyer-Olkin Measure of sampling adequacy (KMO) values for academic burnout, goal orientation, self-regulatory, and academic passion questionnaires are 0.804, 0.922, 0.955, and 0.902, respectively, which show that the volume of data is suitable for factor analysis. Also, based on the amount of surface covered by chi-square statistics (significance level), Bartlett’s index for all variables and their dimensions was equal to 0.001, which is less than the level of 0.01 and showed that the data have a reasonable correlation. To evaluate the reliability of research questionnaires, Cronbach’s alpha coefficient was used. The results showed that Cronbach’s alpha for academic burnout was 0.779, goal orientation was 0.942, self-regulatory was 0.958, and academic passion was 0.947.

#### Research Data Analysis

To analyze the data in two sections of descriptive statistics and inferential statistics, spss 25 and Amos 24 software were used. First, the descriptive statistics section presented the central indicators and the dispersion of research variables. The results are shown in Table 2.

**TABLE 2:** Central indicators and dispersion of research variables.

Variable	*N*	Mean	Std. deviation	Minimum	Maximum	Skewness	Kurtosis
Academic burnout	234	58.193	9.966	22.00	82.00	0.047	0.350
Goal orientation	234	34.490	12.424	16.00	58.00	0.275	−1.209
Self-Regulatory	234	45.223	14.200	20.00	70.00	−0.386	−1.305
Academic passion	234	40.361	14.671	18.00	70.00	0.259	−1.206

The descriptive statistics in Table 2 show that the average academic burnout, goal orientation, self-regulatory, and academic passion are 58.193, 34.490, 45.223, and 40.361, with standard deviations of 9.966, 12.424, 14.200, and 14.671, respectively. Also, the skewness and kurtosis indices are in the range −2 and 2 and show that the distribution of variables is almost normal.

#### Assumptions of Using the Structural Equation Path Analysis Method

##### Default 1: Kolmogorov–Smirnov Test

To evaluate the normality of the research variables, the Kolmogorov–Smirnov normality test is used, the results of which are as follows:

Based on the results of Table 3, it was observed that the sig value is higher than 0.05, and the assumption of normal data is accepted.

**TABLE 3:** Evaluation of normality of variables by the Kolmogorov–Smirnov test.

Variable	The test statistic (K–S)	Sig.	Result
Academic burnout	0.045	*0.200*	The distribution of the variable is normal
Goal orientation	0.042	*0.200*	The distribution of the variable is normal
Self-Regulatory	0.051	*0.165*	The distribution of the variable is normal
Academic passion	0.035	*0.200*	The distribution of the variable is normal

##### Default 2: Pearson Correlation Test

To investigate the correlation of research variables, the Pearson test was used, the results of which are as follows (Table 4):

**TABLE 4:** Correlation between research variables.

		Academic burnout	Goal orientation	Self-regulatory	Academic passion
Academic burnout	Correlation	1			
	Sig. (2-tailed)	–			
Goal orientation	Correlation	−0.805**	1		
	Sig. (2-tailed)	0.000	–		
Self-regulatory	Correlation	−0.709**	0.697**	1	
	Sig. (2-tailed)	0.000	0.000	–	
Academic passion	Correlation	−0.744**	0.702**	0.672**	1
	Sig. (2-tailed)	0.000	0.000	0.000	–

**Correlation is significant at the 0.01 level (2-tailed).

Pearson test results showed that the correlation coefficient of academic burnout with goal orientation is −0.805, self-regulatory is −0.709, and academic passion is −0.744, which is statistically significant at the level of 0.05%. A negative sign indicates that these variables are inconsistent with academic burnout, and with increasing these variables, academic burnout decreases significantly. The correlation coefficient of goal orientation with self-regulatory variables is 0.697, and academic passion is 0.702, which is statistically significant at the level of 0.05%. A positive sign indicates that these variables are homologous with goal orientation, and with increase in these variables, goal orientation rises significantly. The correlation coefficient of self-regulatory with academic passion is 0.672 and is statistically significant at the level of 0.05%. A positive sign indicates that this variable is in line with the self-regulatory, and with increase in academic passion, self-regulatory increases significantly.

#### The Path Analysis Method With Amos 24 Software Is Used to Investigate the Relationship Between Research Variables

**FIGURE 1:**
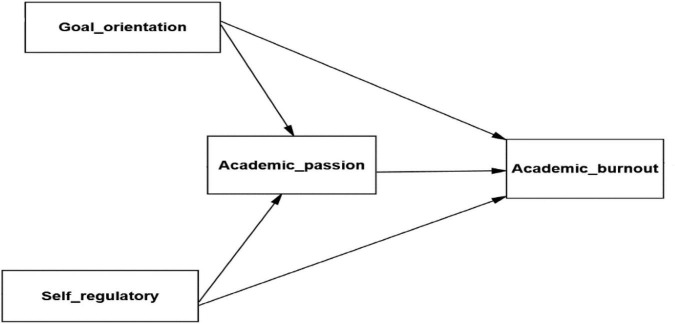
Research model.

**FIGURE 2:**
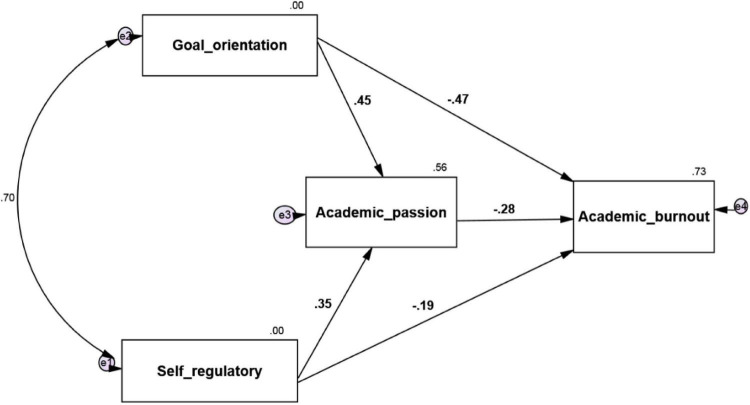
Model fit in standard estimation mode.

**FIGURE 3:**
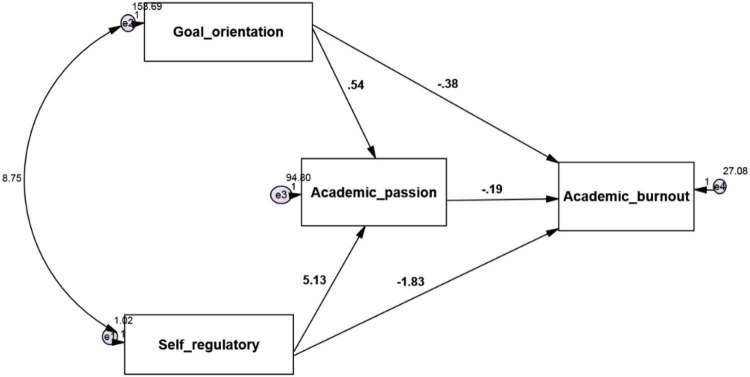
Model fit in non-standard estimation mode.

The research model is as follows (Figures 1–3):

The model was fitted in Amos24 software.

According to the software output, the calculated value of 2χ is equal to 2.867, which is less than three of its degree of freedom, that is, 1. The low value of this index indicates a slight difference between the conceptual model and the observed research data. The RMSEA value is equal to 0.057. The GFI, NFI, IFI, RFI, and CFI are similar to 1.000, 1.000, 1.000, 0.989, and 1.000, respectively, which indicate a high fit. In the following, the research hypotheses are examined.

#### Hypothesis No. 1: Goal Orientation Has a Negative and Significant Effect on the Academic Burnout of English Language Undergraduate Students in Tabriz

The results of this hypothesis are presented in Table 5:

**TABLE 5:** Relationship between goal orientation and academic burnout.

	Path coefficients in the estimation model standard	*t*-value	*P*-value	Situation
Academic burnout ← Goal orientation	−0.475	−8.912	0.001**	Confirmation of the hypothesis

**p < 0.01 at the level of 0.01 is significant.

The results of path analysis in Figure 2 and Table 5 show that the standard coefficient between goal orientation and academic burnout is −0.475, and according to the absolute value of *t*-test statistics, which is equal to 8.912 and higher than 1.96, it can be concluded with a 99% probability of goal orientation. It has a negative and significant effect on academic burnout) β = −0.475, *p*-value = 0.001). In other words, in exchange for increasing one unit of goal orientation, academic burnout decreases by −0.475 units. The coefficient of determination, which is equal to the second power of the path coefficient, is 0.23, which shows that 0.23% of the changes in academic burnout are due to goal orientation.

#### Hypothesis No. 2: Self-Regulation Has a Negative and Significant Effect on the Academic Burnout of English Language Undergraduate Students in Tabriz

The results of this hypothesis are presented in Table 6.

**TABLE 6:** Relationship between self-regulation and academic burnout.

	Path coefficients in the estimation model standard	*t*-value	*P*-value	Situation
Academic burnout ← Self-regulatory	−0.187	−*3.646*	0.001**	Confirmation of the hypothesis

At the level of 0.01 is significant p < 0.01. **At the level of 0.05 is significant p < 0.05.

The results of path analysis in Figure 2 and Table 6 show that the standard coefficient between self-regulation and academic burnout is −0.187, and according to the absolute value of *t*-test statistics, which is equal to 3.464 and higher than 1.96, it can be concluded with 99% probability. Self-regulation has a negative and significant effect on academic burnout (β = −0.187; *p*-value = 0.001). In other words, in exchange for increasing a self-regulatory unit, academic burnout decreases by −0.187 units. The coefficient of determination, which is equal to the square of the coefficients of the path coefficient, is 0.035, which indicates that 3.5% of academic burnout changes are due to self-regulation.

#### Hypothesis No. 3: Goal Orientation Has a Negative and Significant Effect on Academic Burnout With the Mediating Role of the Academic Passion of Undergraduate English Students in Tabriz

A bootstrap test was used to test this hypothesis. The results of this hypothesis are presented in Table 7.

**TABLE 7:** Relationship between goal orientation and academic burnout with the mediating role of academic motivation.

	Path coefficients in the estimation model standard	*P*-value	Situation
Academic burnout ← Academic passion ← Orientation goal	−0.129	0.006**	Confirmation of the hypothesis

**At the level of 0.01 is significant p < 0.01.

The results of path analysis in Figure 2 and Table 7 show that the standard coefficient between goal orientation and academic burnout with the mediating role of academic passion is −0.129, considering its significance level is 0.006 and less than 0.05. It can be concluded that with 99% probability, goal orientation has a negative and significant effect on academic burnout with the mediating role of academic motivation (β = 0.129; *p*-value = 0.006). In other words, in exchange for increasing a unit of goal orientation, academic burnout decreases with the mediating role of academic desire of 0.129 units.

#### Hypothesis No. 4: Self-Regulation Has a Negative and Significant Effect on Academic Burnout With the Mediating Role of the Academic Passion of English Language Undergraduate Students in Tabriz

A bootstrap test was used to test the hypothesis. The results of this hypothesis are presented in Table 8.

**TABLE 8:** Relationship between self-regulation and academic burnout and the mediating role of academic achievement.

	Path coefficients in the estimation model standard	*P*-value	Situation
Academic burnout ← Academic passion ← Self-regulation	−0.101	0.012**	Confirmation of the hypothesis

At the level of 0.01 is significant p < 0.01. **At the level of 0.05 is significant p < 0.05.

The results of path analysis in Figure 2 and Table 8 show that the standard coefficient between self-regulation and academic burnout with the mediating role of academic passion is 0.101. Due to its significance level, equal to 0.012 and less than 0.05, it can be concluded that with a 95% probability, self-regulation has a negative and significant effect on academic burnout with the mediating role of academic motivation (β = −0.101; *p*-value = 0.012). In other words, in exchange for increasing a self-regulatory unit, academic burnout decreases with the mediating role of −0.101 academic achievement.

## Discussion

This research aimed to investigate the mediating role of academic passion in the relationship between goal orientation and academic self-regulation with students’ academic burnout among students at Islamic Azad University, Tabriz. The results of the structural model test showed that the collected data fit well with the conceptual model and the results in general, while confirming the mediating role of academic passion, it showed that goal orientation and self-regulation are negatively correlated with academic burnout. Internal academic passion is negatively correlated with academic apathy, and external passion is positively correlated with academic burnout. Academic passion also mediates the relationship between goal orientation, burnout, and academic self-regulation. Regarding the relationship between academic passion and academic burnout, the literature (Lavasani et al., 2011; Mousavi, 2015; Zaregar et al., 2017) showed a negative correlation between motivation and burnout. The study of Salmela-Aro et al. (2009) also predicted decreased motivation, depression, and burnout. The research findings indicate that goal orientation, academic self-regulation, and dimensions of academic passion explain academic burnout. There is a negative relationship between goal orientation and academic burnout (Sharififard et al., 2014; Jagodics and Szabó, 2022). In their study, they showed that goal orientation had a statistically significant inverse relationship with academic burnout, and when the variables were included in the multivariate regression model, only the relationship between out-of-class performance and academic burnout was substantial. Students with higher goal orientation are less vulnerable to burnout. Pourkarimi and Mobinrahni (2018) showed in their study that there is an important and negative relationship between exposure and academic burnout. Students have more academic orientation and therefore feel less academic burnout) Fernandez-Perez and Martin-Rojas (2022). Consequently, it can be stated that there is a relationship between orientation and academic burnout, which is consistent with the findings of the present study. It can be noted that students with high orientation always have less academic burnout due to their success and increased motivation. When students find the subjects enjoyable and valuable and have an intrinsic motivation to learn, they feel more committed to learning. Their level of academic boredom and apathy is reduced. This finding is consistent with the results of previous research (Barimani et al., 2021).

Based on the self-regulated learning theory and metacognitive processes, students’ procedures constitute self-regulation (Duru and Balkıs, 2017; Anthonysamy and Choo, 2021; Öztürk and Çakıroğlu, 2021). Based on Zimmerman’s (2008) theory, self-regulatory learning strategies include a subset of (1) behavioral self-regulation, (2) motivational self-regulation, (3) cognitive self-regulation, and (4) metacognitive self-regulation (Nikos and George, 2005). In the present study, academic self-regulation was negatively correlated with academic burnout, which is consistent with previous studies (Anthonysamy and Choo, 2021; Öztürk and Çakıroğlu, 2021). This suggests that the dimensions of academic motivation significantly mediate the relationship between academic self-regulation and academic self-efficacy with academic burnout. In explaining this finding, it can be said that when learners use self-regulated learning strategies, they will not only understand the subjects well, but also become motivated and responsible, active and interested in their studies, and can make significant progress in their studies.

Findings showed that academic passion plays a mediating role in the relationship between goal orientation variables and academic self-regulation. These findings are consistent with the results of research by Seif and Ali Akbar (2003), Akhlaghi et al. (2015), and Zhao et al. (2021). In explaining these findings, it can be acknowledged that academic passion indicates the strength of behavior, emotional, and cognitive quality of students’ participation during the activity for learning. Academic passion is an essential mediator in academic self-regulation (Abbasi and Pirani (2015). Also, goal orientation in students can lead to more enthusiasm and, as a result, more learning and better progress. People orient themselves to make ethical decisions, are highly creative, have a participatory spirit, desire to learn, and be entrepreneurial. Factors affecting academic achievement are consistent with the study of Sharifa based on the characteristics of a professor, Zahed et al. (2013) based on the scales of desire for school, Khoroushi et al. (2014) about the relationship between emotional and cognitive desire with students’ self-regulation, Khodai based on predictors of academic desire, Teimouri et al. (2020) based on academic self-concept and desire for school, and Aghamirzayi and Salehi Omran (2013) based on factors affecting students’ academic achievement.

The desire to learn is vital to the extent that it is considered an important learning precursor. On the other hand, more academically, successful students feel competent. They have higher self-regulation because of this sense of adequacy in specific assignments, more interest in the task, an inner desire to master it, and a deep understanding of the subject. Therefore, these students are more likely to pursue the goal of dominating a tendency. By contrast, students who believe in low self-regulation and believe that they cannot perform a particular course’s activities or assignments are more likely to get a passing grade in that course. These students avoid appearing incompetent in the eyes of others. Their main goal is simply to prevent failure and unfavorable judgment. Because they study with external motivation, they only memorize the lessons and do not use advanced and deep cognitive strategies.

## Conclusion

The purpose of the current study was to investigate academic passion as an essential element in the study that can be guaranteed by strengthening and improving students’ academic success. This study showed that goal orientation has a negative and significant effect on the academic burnout of English language undergraduate students; self-regulation has a negative and significant effect on the academic burnout of English language undergraduate students; goal orientation has a negative and significant effect on academic burnout with the mediating role of the academic passion of undergraduate English students, and self-regulation has a negative and significant effect on academic burnout with the mediating role of the academic passion of English language undergraduate students. Further experimental investigations are needed to estimate academic passion, self-regulation, goal orientation, and academic burnout. Considering that the structural equation modeling method has been used to evaluate the suitability of the proposed model, the conclusion of cause and effect must be done with caution.

### Implication, Suggestion, and Limitation

The small study sample of students was a limitation of the present study, which should be observed in generalizing the results of caution. However, based on the appropriateness of the conceptual model in the present study, it is suggested that professors try to strengthen the inner motivation among the students and also make the academic results meaningful for the students by providing accurate and appropriate feedback, they establish a relationship between the amount of effort, the amount of questioning, the amount of participation of students and their successes, which leads to an increase in academic orientation and academic self-regulation, and as a result, reducing academic burnout. According to the findings of the present study, it is suggested that future researchers should investigate the role of gender modulators in the relations of the present conceptual model or other variables, such as the theory of learning strategies and academic help-seeking variables, in the theoretical model of the present study. Finally, it is suggested to those in charge of education that they hold formal and informal training courses, such as workshops, regarding the factors involved in students’ academic burnout, to provide a basis for students’ promotion.

## Data Availability Statement

The raw data supporting the conclusions of this article will be made available by the authors, without undue reservation.

## Ethics Statement

Ethical review and approval was not required for the study on human participants in accordance with the local legislation and institutional requirements. Written informed consent from the patients/participants OR patients/participants legal guardian/next of kin was not required to participate in this study in accordance with the national legislation and the institutional requirements.

## Author Contributions

The author confirms being the sole contributor of this work and has approved it for publication.

## Conflict of Interest

The author declares that the research was conducted in the absence of any commercial or financial relationships that could be construed as a potential conflict of interest.

## Publisher’s Note

All claims expressed in this article are solely those of the authors and do not necessarily represent those of their affiliated organizations, or those of the publisher, the editors and the reviewers. Any product that may be evaluated in this article, or claim that may be made by its manufacturer, is not guaranteed or endorsed by the publisher.
